# Forkhead box O-1 regulates the biological behavior of BMP-2-induced human bone mesenchymal stem cells through mitochondrial dynamics and autophagy

**DOI:** 10.17305/bb.2024.10686

**Published:** 2024-09-02

**Authors:** Weijia Feng, Nannan Chen, Ke Chen, Ting Chen

**Affiliations:** 1 Department of Pediatric Orthopaedic, Xinhua Hospital Affiliated to Shanghai Jiaotong University School of Medicine, Shanghai, China; 2 The School of Materials Science and Engineering, Shanghai Jiao Tong University, Shanghai, China; 3 Shanghai Key Laboratory of Materials Laser Processing and Modification, Shanghai Jiao Tong University, Shanghai, China

**Keywords:** Forkhead box O-1, bone morphogenetic protein-2, bone mesenchymal stem cells, mitochondrial dynamics, mitochondrial autophagy

## Abstract

This study explored the mechanism by which forkhead box O-1 (FoxO1) modulates the biological behaviors of bone mesenchymal stem cell (BMSC). Human BMSCs were cultured for seven days in Dulbecco’s modified Eagle medium (DMEM) containing bone morphogenetic protein-2 (BMP-2) and treated with a short hairpin-FoxO1 plasmid. The study assessed cell proliferation, migration, apoptosis, adenosine triphosphate (ATP) levels, mitochondrial DNA (mtDNA) levels, membrane potential (MMP), autophagy, and the levels of FoxO1, apoptosis-associated proteins, osteogenic differentiation-associated proteins, mitochondrial fusion and fission proteins, and mitochondrial autophagy-related proteins. The cells were also treated with the mitochondrial fusion activator MASM7 and the mitochondrial autophagy activator carbonyl cyanide 3-chlorophenylhydrazone (CCCP). The study evaluated whether mitochondrial dynamics and autophagy activation could rescue the FoxO1 knockdown-induced changes in BMSC biological behaviors, mitochondrial dynamics, and mitochondrial autophagy. BMP-2-induced BMSCs exhibited upregulated FoxO1 expression, enhanced proliferation and migration, and induced osteogenic differentiation, while FoxO1 knockdown inhibited BMP-2-induced BMSC proliferation, migration and osteogenic differentiation, increased apoptosis, and affected mitochondrial dynamics and autophagy. Promoting mitochondrial fusion partially reversed the regulatory effects of FoxO1 downregulation on mitochondrial autophagy and the inhibitory effects of FoxO1 silencing on BMP-2-induced BMSC biological behaviors. Activated mitochondrial autophagy facilitated the homeostasis of mitochondrial dynamics and partially counteracted the inhibitory effects of FoxO1 knockdown on BMP-2-induced BMSC biological behaviors. In conclusion, FoxO1 regulates mitochondrial dynamics and autophagy to modulate the osteogenic differentiation of BMP-2-induced human BMSCs.

## Introduction

Bone marrow mesenchymal stem cells (BMSCs) are a prominent source of osteoblasts and critical cells in the bone formation process, with their osteogenic differentiation and proliferation playing a pivotal role in maintaining the dynamic balance or imbalance between osteogenesis and bone resorption [1]. Notably, BMSCs have shown potential for treating diabetes mellitus, osteoporosis, myocardial infarction, osteoarthritis, and Crohn’s disease [2, 3]. Furthermore, it has been suggested that the differentiation capacity of BMSCs is relevant to steroid-induced osteonecrosis of the femoral head [4].

Bone morphogenetic proteins (BMPs), the largest subgroup of signaling ligands in the transforming growth factor-beta superfamily, are involved in the survival, proliferation, and cell fate of multiple cell types, as well as in many pathological conditions [5]. Importantly, BMPs are biologically active molecules capable of inducing new bone formation [6]. They are commonly recognized as powerful growth factors that facilitate cartilage and bone formation via the induction of osteoblastogenesis and chondrogenesis [7]. Additionally, they play an important role in osteoblast differentiation [8]. Among them, upregulation of bone morphogenetic protein-2 (BMP-2) expression has been documented in osteoblasts [9]. Moreover, overexpressed BMP-2 in fibroblasts repairs serious defects in the cranium [10].

The forkhead box O (FoxO) family of transcription factors is widely involved in modulating several significant biological processes, including apoptosis, energy metabolism, DNA damage repair, and oxidative stress [11, 12]. In mammals, this family primarily consists of four protein isoforms: FoxO4, Forkhead box O-1 (FoxO1), FoxO6, and FoxO3a. Except for FoxO6, which is expressed in neural tissues, the other three are expressed in all tissues throughout the body [13]. Additionally, a growing body of evidence suggests that FoxO1 is essential in maintaining bone homeostasis. For instance, in mouse osteoblasts, knocking down FoxO1 expression accelerates osteoblast apoptosis and oxidative stress, whereas upregulating FoxO1 expression limits osteoblast apoptosis and oxidative stress, and expedites their proliferation [14]. Moreover, FoxO1 silencing diminishes the expression levels of osteogenic markers, including alkaline phosphatase (ALP), runt-related transcription factor 2 (Runx2), and osteocalcin [15]. It is suggested that FoxO1 contributes to modulating osteoblast differentiation and proliferation [16].

Mitochondria are highly dynamic organelles that undergo coordinated cycles of fission and fusion, collectively termed mitochondrial dynamics, which maintain mitochondrial distribution, shape, and size [17]. Mitochondria are the major source of reactive oxygen species (ROS), the overproduction of which results in oxidative stress and damages cellular components. An imbalance in mitochondrial dynamics is closely related to oxidative stress [18]. Dynamin-related protein 1 (Drp1), mitofusin 2 (MFN2), and MFN1 are notable proteins involved in mitochondrial fission and fusion, through which AMPKα1 modulates mitochondrial dynamics and mitigates pathological bone loss [19]. Additionally, osteoblast dysfunction induced by H2O2 can be mitigated through regulation of mitochondrial dynamics balance, thereby facilitating cellular ALP expression and osteoblast differentiation [20]. This suggests that regulating the balance of mitochondrial dynamics can promote osteoblast differentiation.

Overall, both mitochondrial dynamics and autophagy contribute to modulating the differentiation of BMSCs. Furthermore, mitochondrial dynamics are also closely associated with mitochondrial autophagy. Degradation of ubiquitinated MFN2 and MFN1 advances mitochondrial autophagy [21]. Moreover, MFN2 and MFN1 ubiquitination relies on the PINK1/Parkin autophagy pathway, and silencing PINK1 enables convergent expression of mitochondrial dynamics [22]. Thus, mitochondrial dynamics and autophagy are able to regulate each other, together maintaining mitochondrial homeostasis. Additionally, the imbalance of mitochondrial fission and fusion, which leads to mitochondrial dysfunction or damage, is a prerequisite for mitochondrial autophagy, suggesting that mitochondrial dynamics play a pivotal role in regulating mitochondrial autophagy [23, 24].

Mitochondrial autophagy sustains cellular and mitochondrial homeostasis by specifically degrading damaged mitochondria [25]. Meanwhile, mitochondrial autophagy plays a crucial role in the differentiation of BMSCs into osteoblasts, and defective mitochondrial autophagy is closely related to impaired osteogenic capacity in senescent BMSCs [26]. Previous studies have shown that activation of mitochondrial autophagy encourages the expression of DNA damage-regulated autophagy modulator 1 (DRAM1), autophagy-related gene 5 (Atg5), and autophagy-related gene 12 (Atg12) [27–29]. Moreover, mitochondrial autophagy is activated through downregulation of LRRc17 expression and upregulation of LC3 and sequestosome-1 (p62) proteins, thereby attenuating mitochondrial dysfunction and facilitating the transition of BMSCs from lipogenic to osteogenic differentiation [30]. Altogether, this evidence suggests that the differentiation of BMSCs is regulated by modulation of mitochondrial autophagy.

Consequently, we hypothesize that FoxO1 promotes osteoblast proliferation and differentiation by regulating mitochondrial dynamics and autophagy. This study aims to explore the mechanism by which FoxO1 regulates mitochondrial dynamics and autophagy, thereby modulating the biological behavior of BMP-2-induced BMSCs.

## Materials and methods

### Cell culture

Human BMSCs were procured from the American Type Culture Collection (PCS-500-012, Manassas, VA, USA). BMSCs were seeded in Dulbecco’s modified Eagle medium (DMEM) (2 × 10^5^ cells/well) containing 100 U/mL penicillin (Gibco, Thermo Fisher Scientific, Waltham, MA, USA) and 10% fetal bovine serum (FBS, Gibco, Thermo Fisher Scientific) in an incubator with 5% CO_2_ at 37 ^∘^C for culture. Flow cytometry was used to identify negative and positive surface markers of BMSCs. Upon reaching 80% confluence, the attached cells were subcultured by trypsinizing with 0.125% trypsin. The P3-P5 generation of BMSCs was obtained and then seeded in DMEM (1.5 × 10^5^ cells/well) containing 10% FBS, 100 U/mL penicillin, 50-µM ascorbic acid, 10-mM β-glycerophosphate, 2-mM L-glutamine, and 100-nM dexamethasone for osteoblast differentiation induction. The cells were cultured in an incubator at 37 ^∘^C with 5% CO_2_ for seven days [31].

### Cell grouping and treatment

Con group: Cultured with BMP-2-free osteoblast differentiation-inducing medium for seven days. BMP-2 group: Cultured with osteoblast differentiation-inducing medium containing 10 ng/mL BMP-2 for seven days. BMP-2 + sh-NC group: Cultured with osteoblast differentiation-inducing medium containing 10 ng/mL BMP-2 for seven days after transfection with short hairpin (sh)-negative control (NC) plasmid. BMP-2 + sh-FoxO1 group: Cultured with osteoblast differentiation-inducing medium containing 10 ng/mL BMP-2 for seven days after transfection with sh-FoxO1 plasmid. BMP-2 + sh-FoxO1 + MASM7 group: Transfected with sh-FoxO1 plasmid and treated with 1 µM of mitochondrial fusion activator MASM7 (MedChemExpress LLC, Monmouth Junction, NJ, USA) [32], followed by culture in osteoblast differentiation-inducing medium containing 10 ng/mL BMP-2 for seven days. BMP-2 + sh-FoxO1 + DMSO I group: Treated with sh-FoxO1 plasmid and an equal amount of dimethyl sulfoxide (DMSO) as MASM7, followed by culture in osteoblast differentiation-inducing medium containing 10 ng/mL BMP-2 for seven days. BMP-2 + sh-FoxO1 + CCCP group: Cultured in osteoblast differentiation-inducing medium containing 10 ng/mL BMP-2 for seven days after treatment with sh-FoxO1 plasmid and 1 µM of mitochondrial autophagy agonist carbonyl cyanide 3-chlorophenylhydrazone (CCCP; MedChemExpress LLC) [33]. BMP-2 + sh-FoxO1 + DMSO II group: Cultured in osteoblast differentiation-inducing medium containing 10 ng/mL BMP-2 for seven days after treatment with sh-FoxO1 plasmid and an equal amount of DMSO as CCCP. BMSCs were transfected with 100-nM sh-NC and sh-FoxO1 plasmids (Takara Biotechnology, Dalian, Liaoning, China) [34] using Lipofectamine™ 2000 reagent (11668500, Thermo Fisher Scientific).

### Cell counting kit-8 (CCK-8) assay

Cells were incubated at 37 ^∘^C for 0, 24, 48, and 72 h, with the addition of 25-µL CCK-8 reagent (CCK-8, Dojindo Molecular Technologies, Kamimashiki-gun, Kumamoto, Japan) for 2 h. A microplate reader (Bio-Rad 680, Bio-Rad, Hercules, CA, USA) was used to measure the optical density (OD) at 450 nm. The experiment was repeated at least three times.

### Wound healing assay

Cells were seeded onto 6-well culture plates (2 × 10^5^ cells/well) and cultured until the wells were confluent. A scratch wound was created vertically on the cell layer using a sterile pipette tip, and the cells were cultured further for 48 h. Images were captured using an inverted phase-contrast microscope (Olympus, Tokyo, Japan). The healing area was calculated with ImageJ (National Institutes of Health, Bethesda, MD, USA). Migration area ═ (initial scratch area - final scratch area) / initial scratch area × 100%.

### Flow cytometry

BMSCs were detached with 0.125% trypsin (Sigma-Aldrich, St. Louis, MO, USA) and centrifuged for 10 min at 500 × *g* at room temperature. After centrifugation, the cells were rinsed three times with phosphate-buffered saline (PBS), with the concentration adjusted to 1 × 10^6^ cells/mL. The cells were incubated for 20 min in 3% bovine serum albumin (Sigma-Aldrich) at 4 ^∘^C to block nonspecific proteins, followed by labeling with mouse anti-CD31 antibody (1:20, ab9498, Abcam, Cambridge, MA, USA), mouse anti-CD45 antibody (1 µg/10^6^ cells, ab8216, Abcam), mouse anti-CD34 antibody (1:50, ab8536, Abcam), mouse anti-CD73 antibody (1:200, ab202122, Abcam), mouse anti-CD44 antibody (1.055 µg/mL, ab264539, Abcam), mouse anti-CD105 antibody (1.055 µg/mL, ab2529, Abcam), and mouse anti-CD90 antibody (1 µg/10^6^ cells, ab23894, Abcam). Subsequently, the BMSCs were incubated at 4 ^∘^C for 20 min in the dark, and then Goat Anti-Mouse IgG H&L (Alexa Fluor® 488) (1:2000, ab150113, Abcam) was added for a 40-min incubation at room temperature. Finally, surface marker protein expression in BMSCs was analyzed by flow cytometry (Aceabio, San Diego, CA, USA). Annexin V-fluorescein isothiocyanate (FITC)/propidium iodide (PI) dual staining was used to assess BMSC apoptosis. Cells were detached with 0.25% trypsin (Procell Life ScienceTechnology Co., Ltd, Wuhan, Hubei, China) and centrifuged. After three washes with PBS, the cells were resuspended in binding buffer. According to the Annexin V-FITC/PI apoptosis detection kit’s instructions (Elabscience Biotechnology, Wuhan, Hubei, China), the cells were incubated with Annexin V-FITC and PI for 15–20 min at room temperature in the dark, followed by apoptosis analysis using a flow cytometer (Aceabio) within an hour.

### Calcium deposition assay

After 21 days of culture in induction medium, calcium deposition was assessed by alizarin red S staining. Cells were fixed with 4% paraformaldehyde (P0099, Beyotime, Shanghai, China) and stained with 2% alizarin red S solution (Sigma-Aldrich) for 10 min at room temperature, followed by five washes with PBS and imaging. For quantitative detection, 10% cetylpyridinium chloride solution (Sigma-Aldrich) was used to destain the wells for 30 min. The OD value at 590 nm was then measured using a microplate reader (Bio-Rad 680, Bio-Rad).

### Determination of relative mitochondrial DNA (mtDNA) copy number

Cytoplasm was separated using a cell mitochondria separation kit (C3601, Beyotime). Briefly, cells (1 × 10^7^) were incubated in 0.1-mL mitochondrial lysis buffer for 10 min and then homogenized 30 times in a Dounce homogenizer. The homogenate was centrifuged at 600 *g* at 4 ^∘^C for 10 min to remove nuclei and unbroken cells, followed by further centrifugation of the supernatant at 12,000 *g* at 4 ^∘^C for 30 min to collect the cytosolic fraction. DNA from cytosolic fractions was extracted using the QIAQuick nucleotide removal kit (28306, QIAGEN, Valencia, CA, USA). The copy number of mtDNA was determined by reverse transcription quantitative polymerase chain reaction (RT-qPCR). Fluorescent labeling was performed using SYBR Green PCR Master Mix (KGA1339-1, Keygen Biotech, Yixing, Jiangsu, China). qPCR was conducted on an ABI PRISM 7900 sequence detection system with SYBR Green II (Takara Bio), under the following conditions: 95 ^∘^C for 5 min, followed by 40 cycles of 95 ^∘^C for 15 s, 60 ^∘^C for 20 s, and 72 ^∘^C for 35 s. GAPDH served as the internal reference, and data were analyzed using 2^−ΔΔCt^ method. Primers were synthesized by Sangon Biotech (Shanghai, China) and are listed in Table 1.

**Table 1:** Primer sequences

**Gene**	**Forward 5′–3′**	**Reverse 5′–3′**
*ND1*	CCCTAAAACCCGCCACATCT	GAGCGATGGTGAGAGCTAAGGT
*GAPDH*	CTCAGACACCATGGGGAAGGTGA	ATGATCTTGAGGCTGTTGTCATA

### Mitochondrial membrane potential (MMP) assay

Changes in MMP were evaluated using an MMP detection kit (C2003S, Beyotime). After adding 0.5 mL of JC-1 staining solution, cells were incubated at 37 ^∘^C for 20 min in an incubator, followed by centrifugation and cell collection. The cells were washed twice with JC-1 staining buffer, resuspended in an appropriate amount of JC-1 staining buffer, and observed under a fluorescence microscope. Fluorescence intensity was recorded by flow cytometry. When MMP was high, JC-1 aggregated in the mitochondrial matrix to form polymers (J-aggregates), emitting orange fluorescence. When MMP was low, JC-1 could not aggregate in the mitochondrial matrix, and JC-1 existed as monomers that produced green fluorescence.

### Adenosine triphosphate (ATP) content detection

Cellular ATP content was assessed using an ATP content assay kit (ml092826, Mlbio, Shanghai, China). After adding acid extract, cells were subjected to ultrasonication for 1 min, followed by centrifugation at 8000 *g* at 4 ^∘^C for 10 min and mixed with an equal volume of alkaline extract. The cells then underwent another centrifugation, and the supernatant was collected. The OD value at 700 nm was measured using a microplate reader (Bio-Rad 680, Bio-Rad) and converted into ATP concentration. The formula for calculating ATP concentration was: ATP content (µmol/mg protein) ═ [C standard tube × (A assay tube-A control tube) ÷ (A standard tube-A blank tube) × V1] ÷ (V1 ÷ Cpr) ═ 2 × (A assay tube-A control tube) ÷ (A standard tube-A blank tube) ÷ Cpr, where C standard tube represents the standard solution concentration (2 µmol/mL); V1 is the sample volume added to the reaction system (0.01 mL); and Cpr is the concentration of sample protein.

### Mitochondrial autophagy analysis

Cells were incubated with 100 nM Mito-Tracker Red (C1035, Beyotime) for 30 min at 37 ^∘^C, followed by three rinses with DMEM, and then cultured with 50-nM Lyso-Tracker Green (C1047, Beyotime) for 30 min at 37 ^∘^C. Fluorescence microscopy (Leica DMI8, Leica, Heidelberg, BW, Germany) was used to capture images, and ImageJ 1.48 software (National Institutes of Health) was employed for cell counting. The mitochondrial autophagy ratio was calculated as the ratio of the number of Mitotracker/Lysotracker co-localizations to the total number of lysosomes.

### Western blot

Cytoplasmic and nuclear proteins of BMSCs were extracted using the cytosolic and cytoplasmic protein extraction kit (P0027, Beyotime), and protein concentration was determined using a bicinchoninic acid protein assay kit (P0011, Beyotime). After electrophoresis, membrane transfer, and blocking, the samples were incubated with primary antibodies overnight at 4 ^∘^C, followed by washing and incubation with a secondary antibody for 1 hour at 37 ^∘^C. Protein bands were detected using a chemiluminescence kit (ECL Plus, Life Technology, Waltham, MA, USA), and grayscale analysis was performed using ImageJ (National Institutes of Health), with β-actin as the internal reference. Antibody information used in this study is presented in Table 2.

**Table 2:** Antibody information

**Antibodies**	**Cat no.**	**Dilution**	**Company**
Bax	ab32503	1: 1000	Abcam
Cleaved caspase-3	ab32042	1: 500	
Bcl-2	ab182858	1: 2000	
ALP	ab229126	1: 500	
Runx2	ab236639	1: 1000	
MFN1	ab221661	1: 1000	
MFN2	ab205236	1: 2000	
Drp1	ab184247	1: 1000	
β-actin	ab8227	1: 2000	
Goat anti-rabbit IgG H&L (HRP)	ab205718	1: 1000	
Atg5	GTX113309	1: 500	Genetex
DRAM1	GTX85483	2 µg/mL	
Atg12	GTX124181	1: 500	
P62	GTX636570	1: 1000	
LC3	GTX00949	1: 500	
FoxO1	GTX110724	1: 500	

ALP: Alkaline phosphatase; Runx2: Runt-related transcription factor 2; MFN2: Mitofusin 2; Drp1: Dynamin-related protein 1; DRAM1: Damage-regulated autophagy modulator 1; Atg5: Autophagy-related gene 5; Atg12: Autophagy-related gene 12; FoxO1: Forkhead box O-1.

### Statistical analysis

All data were statistically analyzed and graphed using GraphPad Prism 8.01 software (GraphPad Software Inc., San Diego, CA, USA). Measurement data were represented as mean ± standard deviation (SD). Comparisons between two groups were performed using an independent sample *t*-test, and comparisons among multiple groups were conducted using one-way analysis of variance (ANOVA), followed by Tukey’s multiple comparison test. A bilateral test was used, and *P* < 0.05 indicated a statistically significant difference.

## Results

### Knockdown of FoxO1 expression repressed BMP-2-induced biological behaviors in human BMSCs

**Figure 1.**
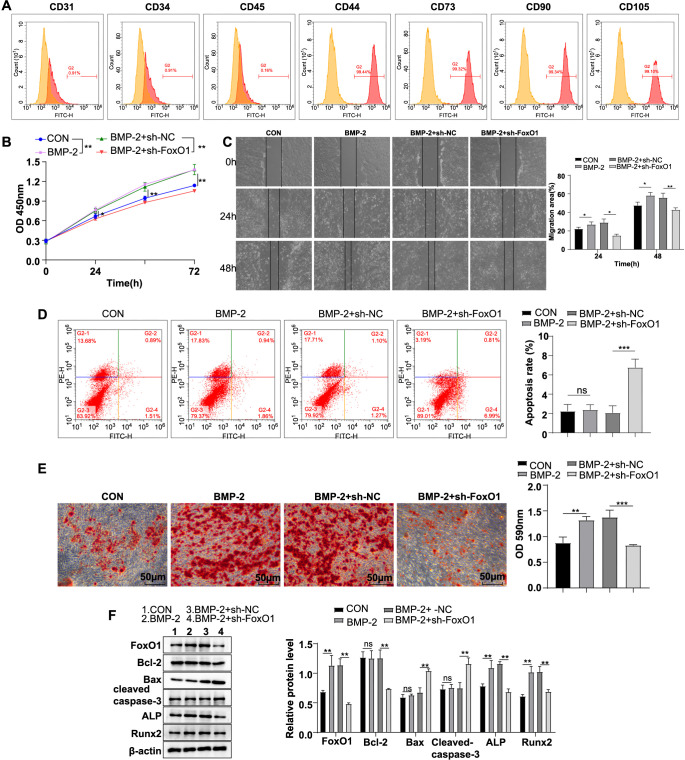
**Knocking down FoxO1 expression restrained BMP-2-induced biological behaviors in human BMSCs.** (A) Flow cytometry was used to identify cell surface markers of BMSCs: Positive markers (CD44, CD73, CD90, and CD105) and negative markers (CD31, CD34, and CD45); (B) CCK-8 assay was conducted to assess cell proliferation; (C) Wound healing assay was performed to evaluate cell migration; (D) Flow cytometry was used to assess cell apoptosis; (E) Alizarin Red S staining was used to detect cellular calcium deposition; (F) Western blot analysis showed levels of cellular FoxO1, apoptosis-associated proteins (Bcl-2, Bax, cleaved caspase-3), and osteogenic differentiation-associated proteins (ALP, Runx2). Experiments were conducted independently in triplicate. Data are expressed as mean ± SD. One-way ANOVA followed by Tukey’s multiple comparison test was used for comparisons. ns *P* > 0.05, * *P* < 0.05, ** *P* < 0.01, *** *P* < 0.001. FoxO1: Forkhead box O-1; BMSC: Bone mesenchymal stem cell; BMP-2: Bone morphogenetic protein-2; CCK-8: Cell counting kit-8; SD: Standard deviation; ANOVA: Analysis of variance; ALP: Alkaline phosphatase; Runx2: Runt-related transcription factor 2.

First, BMSC markers were identified by flow cytometry. The findings showed that the BMSC surface negative markers CD34, CD31, and CD45 all had positive rates of less than 1%, while the positive markers CD44, CD73, CD90, and CD105 exhibited positive rates of more than 99% (Figure 1A), indicating that the BMSCs used were correct. To investigate the regulatory effect of knocking down FoxO1 expression on BMP-2-induced biological behaviors in human BMSCs, the cells were initially cultured with a medium containing BMP-2 for seven days. CCK-8 and wound healing assays revealed that compared with the CON group, cell proliferation and migration in the BMP-2 group were significantly increased (all *P* < 0.05) (Figure 1B and 1C). Flow cytometry indicated no significant difference in cell apoptosis between the BMP-2 group and the CON group (*P* > 0.05) (Figure 1D). Alizarin red S staining showed a marked increase in calcium deposition in the extracellular matrix in the BMP-2 group compared to the CON group (*P* < 0.01) (Figure 1E). Western blot analysis revealed that FoxO1 expression was significantly upregulated in the BMP-2 group compared to the CON group (*P* < 0.01) (Figure 1F). Additionally, Western blot analysis showed no significant differences in Bcl-2, Bax, and cleaved-caspase-3 expression between the BMP-2 group and the CON group (all *P* > 0.05), while ALP and Runx2 protein expression levels were significantly elevated (all *P* < 0.01) (Figure 1F).

Following transfection with the sh-FoxO1 plasmid, cells were cultured in BMP-2-free DMEM for seven days and analyzed experimentally. Compared with the BMP-2 + sh-NC group, the BMP-2 + sh-FoxO1 group displayed notable reductions in proliferative and migratory abilities (all *P* < 0.05), a significant increase in apoptosis (*P* < 0.001), a marked decrease in calcium deposition in the extracellular matrix (*P* < 0.001), noticeable downregulations in the expression levels of FoxO1, Bcl-2, ALP, and Runx2 proteins, as well as significant upregulations in the expression levels of Bax and cleaved caspase-3 proteins (all *P* < 0.01) (Figure 1B–1F).

In conclusion, knockdown of FoxO1 repressed BMP-2-induced biological behaviors in BMSCs.

### Knockdown of FoxO1 expression affected mitochondrial dynamics and autophagy

**Figure 2.**
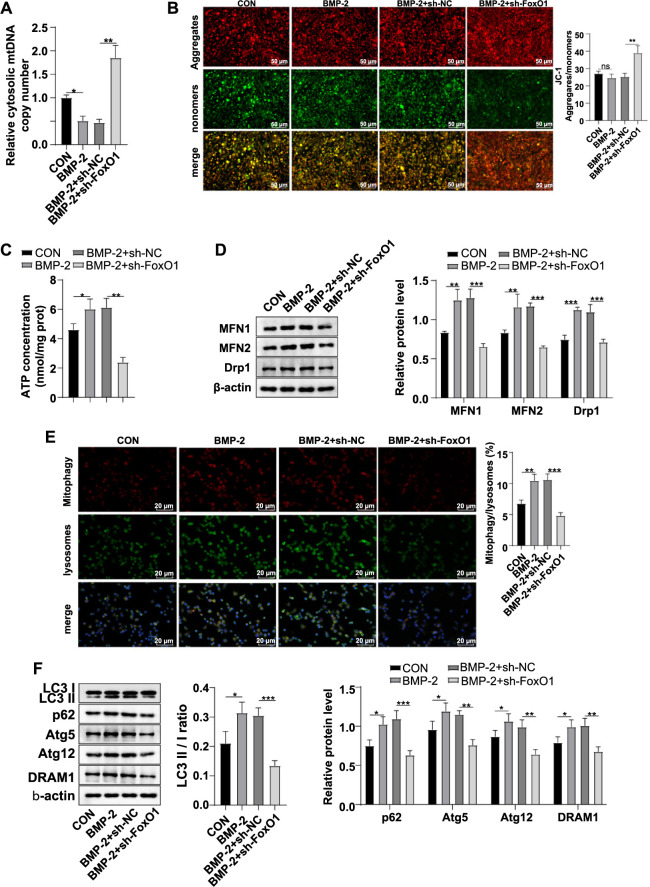
**FoxO1 knockdown affected mitochondrial dynamics and autophagy.** (A) RT-qPCR analysis was used to determine cytoplasmic mtDNA levels; (B) JC-1 probe method was used to detect MMP, where red fluorescence represents the JC-1 polymer, green fluorescence represents the JC-1 monomer, and an elevated polymer/monomer ratio indicates an increased MMP level; (C) Cellular ATP levels; (D) Western blot analysis was performed to examine the mitochondrial fusion and fission protein levels of MFN1, MFN2, and Drp1; (E) Immunofluorescence analysis of mitochondrial autophagy, with Mito-Tracker Red labeling mitochondria and Lyso-Tracker Green labeling lysosomes. The mitochondrial autophagy ratio was calculated as the ratio of Mitotracker/Lysotracker co-localization to the total lysosome count; (F) Western blot analysis of mitochondrial autophagy-related proteins LC3-I, LC3-II, p62, Atg5, Atg12, and DRAM1. Experiments were conducted independently in triplicate, and data are presented as mean ± SD. Comparisons were analyzed by one-way ANOVA, followed by Tukey’s multiple comparison test. ns *P* > 0.05, * *P* < 0.05, ** *P* < 0.01, *** *P* < 0.001. FoxO1: Forkhead box O-1; ATP: Adenosine triphosphate; Drp1: Dynamin-related protein 1; MFN2: Mitofusin 2; DRAM1: Damage-regulated autophagy modulator 1; Atg5: Autophagy-related gene 5; Atg12: Autophagy-related gene 12; mtDNA: Mitochondrial DNA; RT-qPCR: Reverse transcription quantitative polymerase chain reaction; MMP: Membrane potential; SD: Standard deviation; ANOVA: Analysis of variance.

Mitochondrial integrity can be reflected by levels of mtDNA [35]. RT-qPCR analysis showed that the level of cytoplasmic mtDNA was significantly lower in the BMP-2 group than in the CON group (*P* < 0.05), while it was notably higher in the BMP-2 + sh-FoxO1 group than in the BMP-2 + sh-NC group (*P* < 0.01) (Figure 2A). The JC-1 probe assay detecting MMP indicated no significant difference in the MMP level between the BMP-2 group and the CON group (*P* > 0.05), whereas the MMP level was significantly higher in the BMP-2 + sh-FoxO1 group than in the BMP-2 + sh-NC group (*P* < 0.01) (Figure 2B). Additionally, ATP content was significantly increased in the BMP-2 group compared to the CON group (*P* < 0.05), while cytoplasmic ATP content was significantly reduced in the BMP-2 + sh-FoxO1 group compared to the BMP-2 + sh-NC group (*P* < 0.01) (Figure 2C). Western blot analysis showed that the levels of fusion proteins MFN1, MFN2, and fission protein Drp1 were significantly higher in the BMP-2 group than in the CON group (all *P* < 0.01), while the levels of MFN1, MFN2, and Drp1 were significantly reduced in the BMP-2 + sh-FoxO1 group compared to the BMP-2 + sh-NC group (all *P* < 0.001) (Figure 2D). These results indicated that knocking down FoxO1 expression inhibited mitochondrial dynamics. Immunofluorescence showed that mitochondrial autophagy was significantly promoted in the BMP-2 group compared to the CON group (*P* < 0.01) and was significantly limited in the BMP-2 + sh-FoxO1 group compared to the BMP-2 + sh-NC group (*P* < 0.001) (Figure 2E). Moreover, Western blot analysis revealed that the BMP-2 group had a significantly increased LC3-II/I ratio and elevated p62, Atg5, Atg12, and DRAM1 protein levels relative to the CON group (all *P* < 0.05). These levels were significantly reduced in the BMP-2 + sh-FoxO1 group compared to the BMP-2 + sh-NC group (all *P* < 0.01) (Figure 2F). In summary, these findings suggested that the knockdown of FoxO1 impaired mitochondrial autophagy and dynamics.

### Promotion of mitochondrial fusion partially reversed the regulatory effect of knockdown of FoxO1 on mitochondrial autophagy in human BMSCs

**Figure 3.**
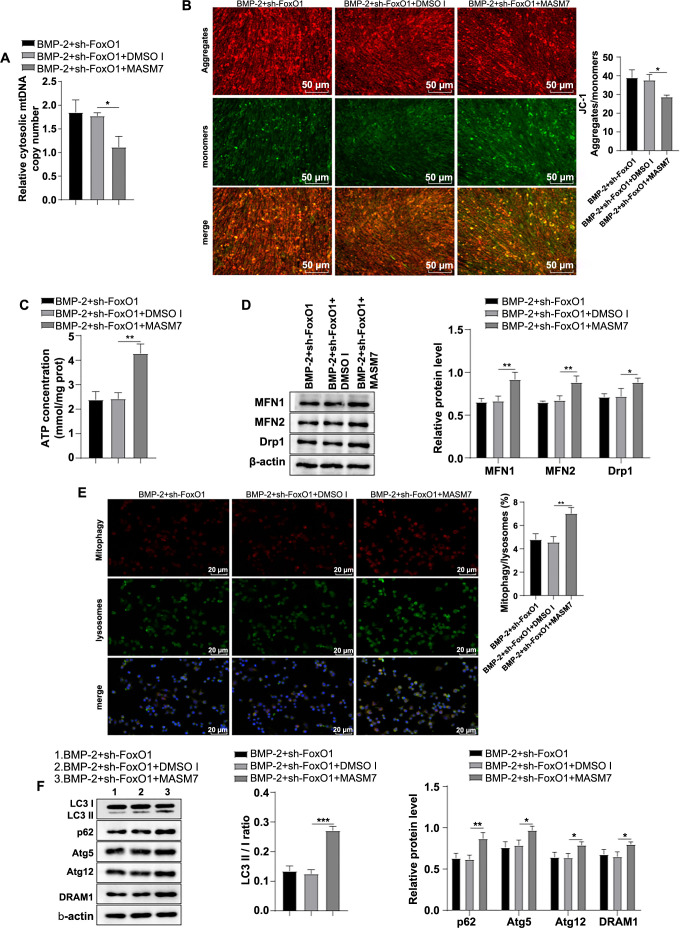
**Facilitating mitochondrial fusion partially abrogated the modulatory role of FoxO1 silencing in mitochondrial autophagy in human BMSCs.** (A) Cytoplasmic mtDNA levels were measured by RT-qPCR; (B) JC-1 probe assay was used to detect MMP; (C) Cellular ATP levels, where red fluorescence represented the JC-1 polymer, green fluorescence represented the JC-1 monomer, and an elevated polymer/monomer ratio indicated an increased MMP level; (D) Western blot was used to measure mitochondrial fusion and fission proteins MFN1, MFN2, and Drp1 levels; (E) Mitochondrial autophagy was assessed by immunofluorescence, with Mito-Tracker Red labeling mitochondria and Lyso-Tracker Green labeling lysosomes; the ratio of Mitotracker/Lysotracker co-localization to the total lysosome count was used to measure the mitochondrial autophagy ratio; (F) Western blot was used to determine levels of mitochondrial autophagy-related proteins LC3-I, LC3-II, p62, Atg5, Atg12, and DRAM1. The cell experiments were repeated three times independently. Data are represented as mean ± SD. One-way ANOVA was applied for comparisons, followed by Tukey’s test. *P < 0.05, ** *P* < 0.01, *** *P* < 0.001. FoxO1: Forkhead box O-1; BMSC: Bone mesenchymal stem cell; ATP: Adenosine triphosphate; Drp1: Dynamin-related protein 1; MFN2: Mitofusin 2; DRAM1: Damage-regulated autophagy modulator 1; Atg5: Autophagy-related gene 5; Atg12: Autophagy-related gene 12; mtDNA: Mitochondrial DNA; RT-qPCR: Reverse transcription quantitative polymerase chain reaction; MMP: Membrane potential; SD: Standard deviation; ANOVA: Analysis of variance.

Next, cells were treated with the sh-FoxO1 plasmid and 1 µM of the mitochondrial fusion activator MASM7, followed by culture in BMP-2 medium for seven days. Compared with the BMP-2 + sh-FoxO1 + DMSO I group, the BMP-2 + sh-FoxO1 + MASM7 group showed a significantly decreased cytoplasmic mtDNA level (*P* < 0.05) (Figure 3A), a notably reduced MMP level (*P* < 0.05) (Figure 3B), a significantly increased ATP content (*P* < 0.01) (Figure 3C), a marked increase in mitochondrial autophagy (*P* < 0.01) (Figure 3E), and significantly elevated LC3-II/I ratio, along with increased levels of MFN1, MFN2, Drp1, p62, Atg5, Atg12, and DRAM1 proteins (all *P* < 0.05) (Figure 3D/3F). These results underscored that promoting mitochondrial fusion partially counteracted the regulatory effect of FoxO1 knockdown on mitochondrial autophagy in human BMSCs.

**Figure 4.**
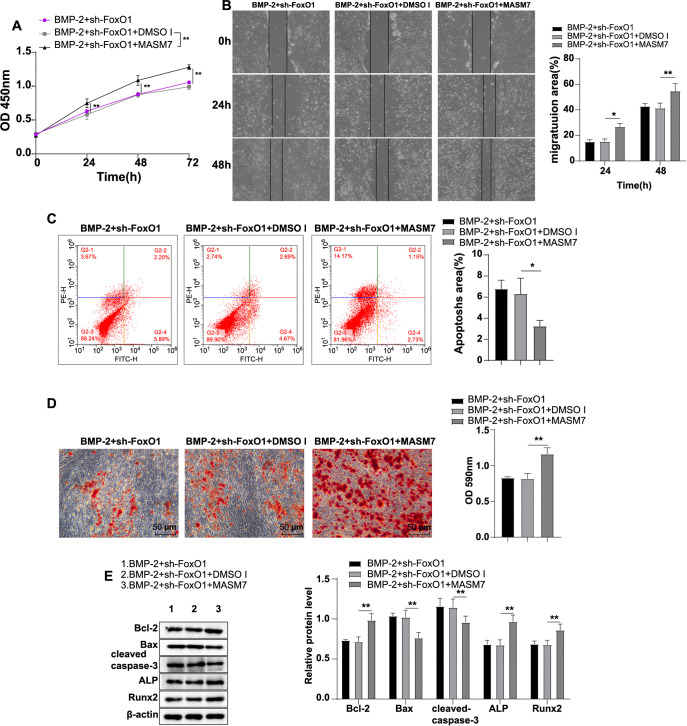
**Mitochondrial fusion promotion partially reversed the suppressive effects of FoxO1 knockdown on BMP-2-induced biological behaviors of human BMSCs.** (A) Cell proliferative ability was assessed by the CCK-8 assay; (B) Cell migratory ability was evaluated by the wound healing assay; (C) Cell apoptosis was assessed by flow cytometry; (D) Alizarin Red S staining was used to detect cellular calcium deposition; (E) Western blot analysis was conducted to determine expression levels of cell FoxO1, apoptosis-related proteins (Bcl-2, Bax, cleaved caspase-3), and osteogenic differentiation-related proteins (ALP, Runx2). The cell experiments were repeated independently three times. Data are expressed as mean ± SD. One-way ANOVA was implemented to analyze comparisons among multiple groups, followed by Tukey’s test, *P < 0.05, ** *P* < 0.01. FoxO1: Forkhead box O-1; BMSC: Bone mesenchymal stem cell; BMP-2: Bone morphogenetic protein-2; CCK-8: Cell counting kit-8; SD: Standard deviation; ANOVA: Analysis of variance; ALP: Alkaline phosphatase; Runx2: Runt-related transcription factor 2.

### Advancing mitochondrial fusion partially abrogated the suppressive effects of FoxO1 silencing on BMP-2-induced biological behaviors of human BMSCs

Subsequently, we examined the biological behaviors of cells in the BMP-2 + sh-FoxO1 + MASM7 group. The results showed significant increases in cell proliferation and migration in the BMP-2 + sh-FoxO1 + MASM7 group relative to the BMP-2 + sh-FoxO1 + DMSO group (all *P* < 0.05) (Figure 4A and 4B), a notable reduction in apoptosis (*P* < 0.01) (Figure 4C), a substantial increase in calcium deposition in the extracellular matrix (*P* < 0.01) (Figure 4D), marked upregulations in the expression levels of cellular Bcl-2, ALP, and Runx2 proteins, and significant downregulations in the expression levels of Bax and cleaved-caspase-3 proteins (all *P* < 0.01) (Figure 4E). These findings indicated that promoting mitochondrial fusion partially mitigated the inhibitory effects of FoxO1 knockdown on BMP-2-induced biological behaviors inhuman BMSCs.

### Activation of mitochondrial autophagy facilitated homeostasis of mitochondrial dynamics

**Figure 5.**
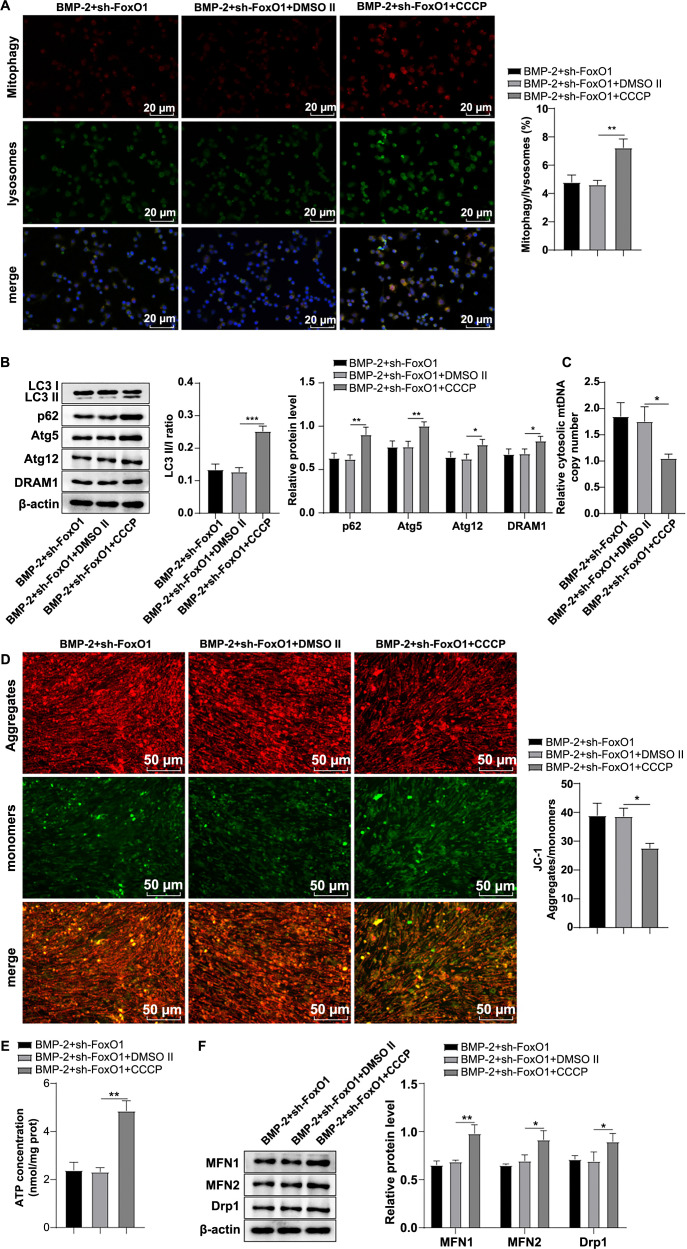
**Activating mitochondrial autophagy encouraged homeostasis of mitochondrial dynamics.** (A) Immunofluorescence detection of mitochondrial autophagy, with Mito-Tracker Red labeling mitochondria and Lyso-Tracker Green labeling lysosomes. The ratio of Mitotracker/Lysotracker co-localization to the total lysosome count was used to measure the mitochondrial autophagy ratio; (B) Western blot detection of mitochondrial autophagy-related protein levels: LC3-I, LC3-II, p62, Atg5, Atg12, and DRAM1; (C) RT-qPCR detection of cytoplasmic mtDNA levels; (D) JC-1 probe method was applied for detecting MMP, where red fluorescence represented the JC-1 polymer, green fluorescence represented the JC-1 monomer, and an elevated polymer/monomer ratio indicated an increased MMP level; (E) Cellular ATP levels; (F) Western blot analysis was used to assess mitochondrial fusion and fission protein levels of MFN1, MFN2, and Drp1. The cell experiments were repeated independently three times. Data are expressed as mean ± SD. Comparisons were analyzed by one-way ANOVA, with post-hoc tests performed using Tukey’s multiple comparison test. *P < 0.05, ** *P* < 0.01, *** *P* < 0.001. ATP: Adenosine triphosphate; Drp1: Dynamin-related protein 1; MFN2: Mitofusin 2; DRAM1: Damage-regulated autophagy modulator 1; Atg5: Autophagy-related gene 5; Atg12: Autophagy-related gene 12; mtDNA: Mitochondrial DNA; RT-qPCR: Reverse transcription quantitative polymerase chain reaction; MMP: Membrane potential; SD: Standard deviation; ANOVA: Analysis of variance.

To further assess the regulatory effect of FoxO1 on mitochondrial autophagy in human BMSCs, we treated FoxO1-silenced human BMSCs with the mitochondrial autophagy activator CCCP. The results showed a significant increase in mitochondrial autophagy in the BMP-2 + sh-FoxO1 + CCCP group compared to the BMP-2 + sh-FoxO1 + DMSO II group (*P* < 0.01) (Figure 5A), along with notable increases in the LC3-II/I ratio and the levels of MFN1, MFN2, Drp1, p62, Atg5, Atg12, and DRAM1 proteins (all *P* < 0.05) (Figure 5B/5F). Furthermore, there was a significant decrease in cytoplasmic mtDNA level (*P* < 0.05) (Figure 5C), a marked reduction in the MMP level (*P* < 0.05) (Figure 5D), and a significant increase in ATP content (*P* < 0.01) (Figure 5E). These results demonstrated that activation of mitochondrial autophagy partially attenuated the FoxO1 knockdown-mediated imbalance of BMP-2-induced mitochondrial dynamics in human BMSCs, thereby playing a crucial role in facilitating mitochondrial dynamicshomeostasis.

### Activation of mitochondrial autophagy partially nullified the suppression effects of FoxO1 knockdown on BMP-2-induced biological behaviors in human BMSCs

**Figure 6.**
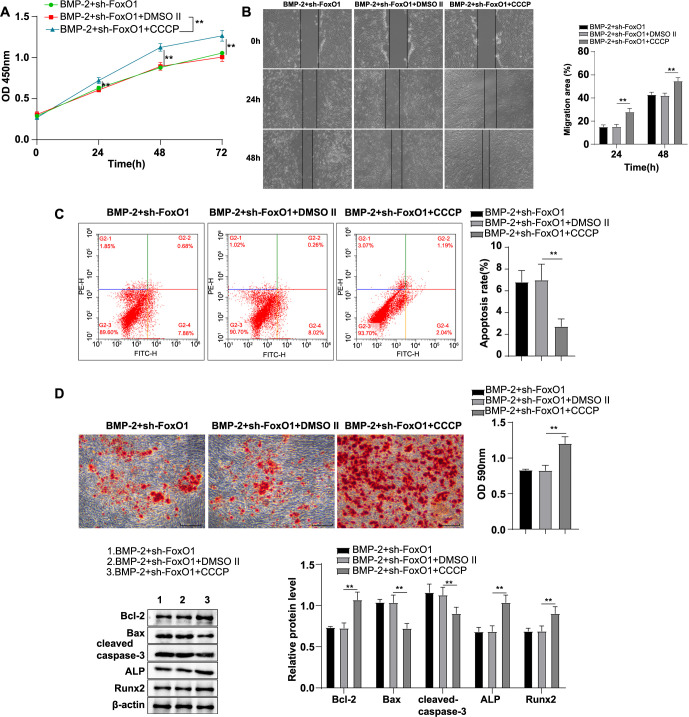
**Activation of mitochondrial autophagy partially abolished the inhibitory effects of FoxO1 knockdown on BMP-2-induced biological behaviors of human BMSCs.** (A) CCK-8 assay was used to evaluate cell proliferative ability; (B) Wound healing assay was conducted to assess cell migratory ability; (C) Flow cytometry was used to evaluate apoptosis; (D) Alizarin Red S staining was used to detect cell calcium deposition; (E) Levels of cell FoxO1, apoptosis-associated proteins (Bcl-2, Bax, cleaved caspase-3), and osteogenic differentiation-associated proteins (ALP, Runx2) were measured by Western blot. The cell experiments were repeated independently three times. Data are represented as mean ± SD, with one-way ANOVA employed for intergroup comparisons, followed by Tukey’s multiple comparison test. * *P* < 0.05, ** *P* < 0.01. FoxO1: Forkhead box O-1; BMSC: Bone mesenchymal stem cell; BMP-2: Bone morphogenetic protein-2; CCK-8: Cell counting kit-8; SD: Standard deviation; ANOVA: Analysis of variance; ALP: Alkaline phosphatase; Runx2: Runt-related transcription factor 2.

Finally, we investigated the effect of activating mitochondrial autophagy on the inhibitory effects of FoxO1 knockdown on BMP-2-induced biological behaviors in human BMSCs. Compared with the BMP-2 + sh-FoxO1 + DMSO II group, cell proliferation and migration were significantly enhanced in the BMP-2 + sh-FoxO1 + CCCP group (all *P* < 0.01) (Figure 6A and 6B), apoptosis was significantly reduced (*P* < 0.01) (Figure 6C), calcium deposition in the extracellular matrix was markedly increased (*P* < 0.01) (Figure 6D), the expression levels of cellular Bcl-2, ALP, and Runx2 proteins were significantly upregulated, and the expression levels of Bax and cleaved-caspase-3 proteins were significantly downregulated (all *P* < 0.01) (Figure 6E). These results indicated that activation of mitochondrial autophagy partially counteracted the inhibitory effects of FoxO1 knockdown on BMP-2-induced biological behaviors in human BMSCs.

## Discussion

BMSCs contribute to maintaining homeostasis in the body by regenerating and repairing aged and damaged tissues and may hold significant therapeutic potential for preventing and treating diseases with pro-inflammatory pathogenesis [1]. Evidence suggests that FoxO transcription factors protect against oxidative stress by activating genes involved in apoptosis and free radical scavenging [14]. Furthermore, the osteogenic differentiation of BMSCs is regulated through the modulation of FOXO1 [36]. In line with this, our findings highlighted that FoxO1 regulates BMP-2-induced biological behaviors in BMSCs by modulating mitochondrial dynamics and autophagy.

FoxO1, highly expressed in the skeleton, is a major regulator of osteoblastic function and bone metabolism [14]. Additionally, FoxO1 influences bone metabolism by acting on osteoblasts and affects bone homeostasis through modulation of the redox balance [37]. FOXO1 has been shown to play a critical role in restraining proliferation and promoting osteoblast differentiation in differentiating cells [16]. Moreover, it is an important transcription factor that interacts with the RUNX2 promoter, expediting osteoblast differentiation and differentiation-related proliferation interruption [16]. Accordingly, we observed reduced cell proliferation and migration, increased apoptosis, decreased calcium deposition in the extracellular matrix, downregulated expression of Bcl-2, ALP, and Runx2 proteins, and upregulated expression of Bax and cleaved caspase-3 proteins following FoxO1 knockdown. In contrast, further treatment with the mitochondrial fusion activator MASM7 or the mitochondrial autophagy activator CCCP reversed these trends. Consistent with our findings, FoxO1 has been demonstrated to modulate BMSC biological behaviors [14]. FoxO1 knockdown reduces bone formation rate and bone volume in FoxO1OB-/- mice, possibly due to a reduced number of osteoblasts; additionally, FoxO1 knockdown enhances caspase-3 activity associated with apoptosis in osteoblasts and suppresses the expression of osteogenic markers (Runx2, ALP), thereby inhibiting osteoblast differentiation [37]. Knocking down FoxO1 also decreases Runx2 and ALP expression in precursor mouse osteoblasts [15]. Collectively, FoxO1 silencing limited BMP-2-induced biological behaviors in BMSCs, while the inhibitory effects were partially counteracted by promoting mitochondrial fusion or activating mitochondrial autophagy.

Mitochondrial dynamics likely play a critical role in determining the differentiation trajectory of BMSCs [38]. PKM2 promotes adipogenesis and inhibits osteogenesis by regulating mitochondrial fusion and fission as well as the β-cyclin signaling pathway [39]. Mitochondrial dynamics can regulate mitochondrial autophagy, which is essential for BMSC differentiation into osteoblasts [23, 24, 26]. Recent studies have reported that promoting mitochondrial fission facilitates osteogenesis, while promoting mitochondrial fusion negatively affects bone formation [40, 41]. Importantly, FoxO1/3 gene silencing via shFoxO1/3 in organoids enhances mitochondrial fission in cells, indicating that FoxO1 is involved in regulating mitochondrial dynamics [42]. Furthermore, FOXO1 promotes the expression of multiple genes associated with autophagy, and its post-translational modifications are essential for initiating the autophagic process [43]. Notably, mitochondrial autophagy can be promoted through the SIRT1/FoxO1 pathway to alleviate apoptosis and mitochondrial damage in bovine ovarian granulosa cells caused by oxidative stress [44]. Similarly, our findings revealed altered mitochondrial dynamics, as shown by elevated mtDNA levels and MMP, decreased ATP content, and reduced levels of MFN1, MFN2, and Drp1, as well as impaired mitochondrial autophagy, evidenced by a reduction in mitochondrial autophagy and decreased LC3-II/I ratio, Atg5, p62, DRAM1, and Atg12 protein levels following FoxO1 silencing in BMP-2-induced BMSCs. However, these trends were partially reversed by further treatment with MASM7 or CCCP. These results suggest that promoting mitochondrial fusion or activating mitochondrial autophagy partially counteracted the regulatory effects of FoxO1 knockdown on mitochondrial dynamics and autophagy in human BMSCs.

## Conclusion

In summary, this study confirms that FoxO1 regulates mitochondrial dynamics and autophagy to control BMP-2-induced biological behaviors in human BMSCs. However, this study has limitations. The focus is solely on the regulatory effects of FoxO1 on mitochondrial dynamics, autophagy, and biological behaviors in human BMSCs, without specifying the exact target pathway of action. Further research is needed to explore the specific pathway through which FoxO1 regulates mitochondrial dynamics, autophagy, and biological behaviors in human BMSCs.

## Data Availability

All the data generated or analyzed during this study are included in this published article.
